# Weight concern and desire for weight loss support in adolescents: Results from a large cross‐sectional school survey

**DOI:** 10.1002/jcv2.70124

**Published:** 2026-04-16

**Authors:** Melissa Little, Mina Fazel, Susan A. Jebb, Sarah Wane, Oxwell Study Team, Paul Aveyard

**Affiliations:** ^1^ Department of Primary Care University of Oxford Oxford UK; ^2^ Deparment of Psychiatry University of Oxford Oxford UK

**Keywords:** adolescence, adolescents, mental health, paediatric obesity, weight concern, weight support

## Abstract

**Background:**

Excess weight and weight concern among adolescents can lead to adverse physical and psychosocial health consequences. This study aims to examine levels of weight concern, desire for weight support, and their associations in adolescents.

**Methods:**

A cross‐sectional survey was conducted in 80 English secondary schools as part of the 2023 OxWell Student Survey. Weight concern was measured on a 4‐point scale, and desire for weight support with yes/no/I don't know response options. Data was analysed using mixed‐effects logistic regression adjusting for gender, year group, ethnicity, and deprivation, with school ID as a random effect.

**Results:**

Of 34,245 respondents (aged 11–18 years), 41% reported some level of weight concern, which was more common among older adolescents, those of White ethnicity, and those from deprived backgrounds. Girls, gender non‐disclosing, and gender diverse young people were significantly more likely than boys to report weight concern with gender diverse young people having eight times higher odds of weight concern. Thirty percent of young people expressed a desire for weight management support, with modest increases by age but minimal variation by ethnicity or deprivation. Weight concern was strongly associated with desire for support.

**Conclusion:**

This study found that weight concern is widespread among UK adolescents, with around one in three expressing a desire for weight management support. These findings reveal a substantial unmet need, as current services targeting only those with overweight or obesity fail to address the broader weight‐related distress experienced by many young people.

## Key Points

**What's known?**

- Excess weight and weight concern among adolescents can lead to adverse mental, physical and ‐social health consequences.

**What's new?**

- Weight concern is widespread among UK adolescents, particularly among girls, gender‐diverse young people, and those from White ethnic backgrounds.
- Around one in three adolescents expressed a desire for weight management support
- Desire for support is strongly linked to adolescents' concerns about their weight, highlighting a clear connection between weight perception and help‐seeking behaviour.

**What's relevant?**

- These findings reveal a substantial unmet need, as current services targeting only those with overweight or obesity fail to address the broader weight‐related distress experienced by many young people.

## INTRODUCTION

Excess weight in young people tracks into adulthood and is one of the leading contributors to ill‐health (Viner et al., 2017). Currently, approximately one third of 11‐year olds in England are classified as above a healthy weight (Digital, 2024). Excess weight during adolescence is associated with a range of adverse physical health outcomes, including metabolic syndrome, sleep disturbances, and pubertal irregularities (Jebeile et al., 2022; Kansra et al., 2020). It is also associated with mental health challenges such as anxiety and depression (Blundell et al., 2024; Marmorstein et al., 2014), and psychosocial difficulties such as social isolation and bullying (Jebeile et al., 2021).

Weight concern, often conceptualised as body dissatisfaction, is highly prevalent among adolescents, with up to one third of girls and one quarter of boys reporting dissatisfaction with their weight or shape (Al Sabbah et al., 2009; Ferreiro et al., 2014). Evidence suggests that weight concern is associated with poor mental health, irrespective of actual weight status (Bornioli et al., 2020; Carapeto et al., 2020) with internalised pressure to achieve an idealised body shape linked to depressive symptoms and broader psychological distress (Blundell et al., 2024; Bornioli et al., 2020). A growing body of research indicates that societal norms, media portrayals, and peer influence play a role in shaping adolescents' perceptions of body image and contributing to the development of weight‐related concerns (Kelly et al., 2018; Vuong et al., 2021).

Despite strong societal pressure to attain an ‘ideal’ body weight, there is limited research examining whether weight concern translates into a desire for structured weight loss support. A 2022 study (Ahmad et al., 2022) reported that weight loss attempts were common among adolescents, including those within a healthy weight range. However, it remains unclear how many young people desire formal support to manage their weight and how weight and shape concern may influence this desire for weight loss support. Adolescents' willingness to seek or engage with weight management services may be influenced by several factors, including stigma, shame, and previous negative experiences with healthcare providers (Little et al., 2025; Puhl & Lessard, 2020). These considerations complicate the relationship between weight concern and help‐seeking behaviour.

This analysis uses a large school‐based dataset to assess the proportion of adolescents with weight and shape concern, and how these concerns relate to the desire for weight loss support. We examine how these patterns vary across demographic factors including age, gender, ethnicity, and socioeconomic status. Furthermore, we investigate whether weight concern independently predicts desire for weight‐related support, and whether this relationship is moderated by demographic characteristics. This analysis seeks to address a key evidence gap in adolescents' support preferences, helping stakeholders understand the current landscape and to inform future interventions and public health strategy.

## METHODS

### Study design and procedure

The OxWell Student Survey asked students a substantial number of questions, covering factors related to wellbeing, school/college experience, mental health, and experiences of accessing mental health support. Informed consent was collected following which participants could access the survey. The data were collected without personal identifiers to encourage accurate responses. Full survey details are provided elsewhere (Mansfield et al., 2021).

Participation in the OxWell Survey was voluntary, and participants did not receive incentives to participate. The survey was online and conducted during school hours. The study was approved by the Research Ethics Committee at the University of Oxford (R62366). This analysis looks only at secondary school data and focuses on two key questions: (1) To what extent do you worry about: my body shape and/or weight, (2) Would you like help to lose weight? No weight data were collected.

### Analysis

#### Frequency analysis

The outcome variable of ‘body shape or weight concern’ was derived from the question ‘To what extent do you worry about: my body shape or weight?’. Participants provided a scaled answer as follows: not at all worried (0), not very worried (1), quite worried (2), worried (3), extremely worried (4).

The ‘desire for weight support’ outcome variable was derived from the question, ‘Would you like help to lose weight?’. Possible answers were yes, no, I don't know and prefer not to say. A no‐response answer was also given to participants who saw the question but chose not to respond.

The proportions answering for each of the above outcome variables was calculated both overall and by demographic factors as follows:

##### Year group

The survey asked, ‘What is your year group?’ Possible answers ranged from year 7 to year 13.

##### Gender

The survey asked ‘What is your gender?’ Possible answers were female, male, other, and prefer not to say and were recorded as female, male, gender diverse, and gender non‐disclosing respectively (Soneson et al., 2024). Anyone who saw the question but chose not to respond was put into the gender non‐disclosing group.

##### Ethnicity

The survey asked, ‘What is your ethnic group?’ Answers were presented as 18 options and grouped according to the 2021 census classification 6a (National & 2021, 2023). A no‐response category was also used in the analysis for those who saw the question but chose not to respond.

##### Deprivation

Deprivatation was calculated according to previously published research (Bignardi, 2025) using the following eight questions (Box 1) each scored by participants as 1 (never or hardly ever), 2 (some of the time) or 3 (often). Once non‐respondents were filtered out, the mean score of these eight questions was taken to give each participant an overall deprivation score.

**BOX 1 Questions used to calculate deprivation**

**Table:** 

1	I worry about not having enough money for the things my family needs, for example, food, bills, electric or gas
2	My family uses food banks
3	The house I live in is cold and/ damp
4	At school, I am unable to afford the right uniform, games kit, books, equipment, or go on trips
5	At school, I am unable to afford to eat
6	At home, I do not have enough space to do things like homework or chill out
7	At home, I have no internet access or poor internet access
8	At home, I go to bed hungry because there is not enough food in the house

#### Regression analysis

We calculated the odds ratio for each predictor variable associated with reporting one level higher of concern about their weight using ordinal logistic regression, mutually adjusted for all other independent variables. Answers of 0–4 were included (the answer of no response was removed). We used binary logistic regression to calculate the odds ratio for desire for weight loss support versus not wanting it, excluding other response options (‘not sure’ and ‘prefer not to say’). Again, all models were mutually adjusted for the included predictors. The independent/predictor variables used were year group, gender, ethnicity and deprivation. For the regression analysis, the deprivation score was converted to an ordinal variable with categories of 1–1.99, 2–2.99 or >3 dependant on the average score of the 8 deprivation questions. School ID was used as a random effects variable to account for clustering within schools. This accounts for any between‐school variation that could affect the outcome variable.

To examine whether weight concern was associated with desire for weight support, a logistic regression model was fitted with desire for weight support as the outcome variable. Weight concern was included as the primary predictor, alongside demographic variables (gender, ethnicity, year group, and deprivation), which were included as covariates. School ID for individual schools was included as a random effect to account for clustering within schools. An initial model was run without interaction terms to assess the main effects of demographic variables and weight concern on desire for weight support, independent of other variables. To examine whether the association between weight concern and desire for support varied by demographic subgroup, four separate models were run, each including an interaction term between weight concern and one demographic variable (e.g., gender, ethnicity). Results are reported as odds ratios with corresponding confidence intervals and *p*‐values.

## RESULTS

### Study population

**FIGURE 1:**
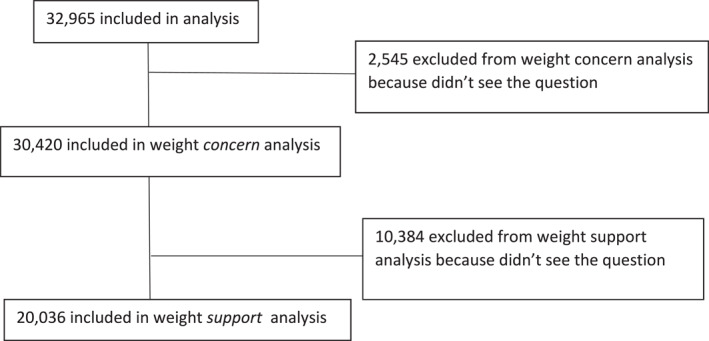
Flow chart of participants.

Data for this analysis were collected from February–March 2023 and 43,734 students participated from 105 primary schools, 70 secondary schools and 10 further education colleges primarily across four different regions in England—Berkshire, Liverpool, Milton Keynes, and Oxfordshire. A total of 34,245 secondary school students participated in the 2023 OxWell Survey. Of those, 1280 (3.7%) were excluded as they didn't complete the consent correctly. A further 2545 (7.4%) were excluded from the weight *concern* analysis as they did not get far enough in the survey to answer that question, and an additional 10,384 (30.3%) were excluded in the weight *support* analysis as they did not get sufficiently far in the survey to see that question (Figure 1).

Table 1 displays the demographic factors for each analysis group. The groups were demographically similar with fewer participants in the second analysis.

**TABLE 1:** Participant group demographics.

	Total (32,965)		Weight concern analysis (30,420)		Weight support analysis (20,036)	
	(*N*)	(%)	(*N*)	(%)	(*N*)	(%)
Year group						
7	6900	20.93	6421	21.11	4070	20.31
8	6259	18.99	5925	19.48	3997	19.95
9	6002	18.21	5596	18.40	3762	18.78
10	4693	14.24	4302	14.14	2758	13.77
11	4480	13.59	4072	13.39	2728	13.62
12	2810	8.52	2507	8.24	1661	8.29
13	1821	5.52	1597	5.25	1060	5.29
Total	32,965	100	30,420	100.00	20,036	100.00
Ethnicity						
White (aggregated)	17,148	52.02	16,046	52.75	10,784	53.82
Mixed (aggregated)	1812	5.50	1687	5.55	1113	5.56
Asian (aggregated)	4546	13.79	4233	13.92	2946	14.70
Black (aggregated)	1621	4.92	1454	4.78	862	4.30
Arab	601	1.82	567	1.86	331	1.65
Other	733	2.22	678	2.23	410	2.05
No response	6504	19.73	5755	18.92	3590	17.92
Total	32,965	100	30,420	100.00	20,036	100.00
Gender						
Girl	16,045	48.67	14,937	49.10	10,391	51.86
Boy	15,038	45.62	13,784	45.31	8554	42.69
Gender diverse	414	1.26	389	1.28	303	1.51
Gender non‐disclosing	1468	4.45	1310	4.31	788	3.93
Total	32,965	100	30,420	100.00	20,036	100.00
Mean deprivation	1.16 (SD = 0.29)		1.16 (SD = 0.29)		1.15 (SD = 0.27)	

### Weight concern

**FIGURE 2:**
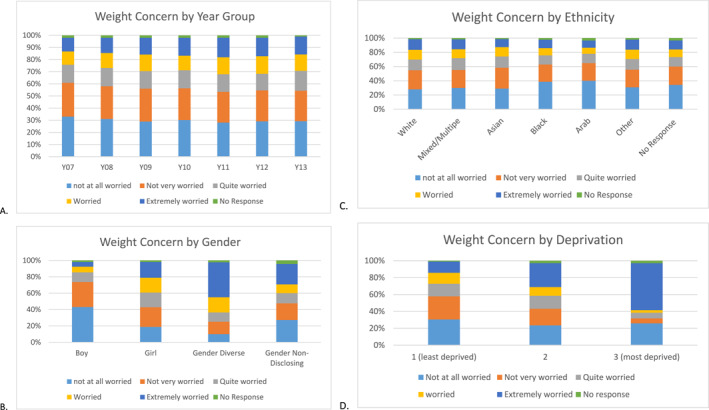
Proportion with weight concern by (A) year group. (B) Gender. (C) Ethnicity. (D) Level of deprivation.

Of the 30,420 young people included in this analysis, 41.2% were either quite worried, worried or extremely worried about their weight; of which 13.7% were extremely worried. Breakdown by year group, gender, ethnicity and level of deprivation is shown in Figure 2. Compared to boys, concern was higher among girls (OR 3.56 95% CI [3.36, 3.78], *p*‐value < 0.001), gender non‐disclosing (OR 3.70 95% CI [3.23, 4.25], *p*‐value < 0.001) and gender diverse students (OR 8.25 95% CI [6.78, 10.23], *p*‐value < 0.001). Weight concern increased slightly with age, with year 8 (age 12–13) students (OR 1.14 95% CI [1.07, 1.21], *p*‐value < 0.001) showing less risk of weight concern than year 13 (age 17–18) students (OR 1.42 95% CI [1.26, 1.60], *p*‐value < 0.001).

Compared with people from the White ethnic group, people from Asian, Black, and Arab ethnic groups had lower weight concern.

### Desire for weight support

**FIGURE 3:**
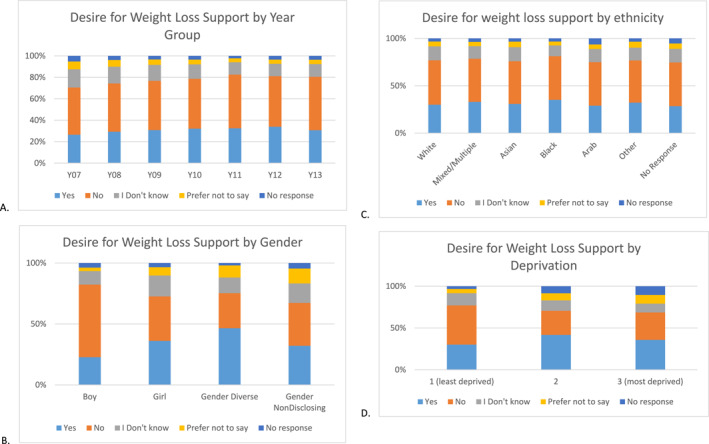
Proportion with weight concern by (A) year group. (B) Gender. (C) Ethnicity. (D) Level of deprivation.

Of the 20,036 participants included in the weight support analysis just under one third expressed a desire for weight loss support, just under half wanted no support and 14% did not know. Breakdown by year group, gender, ethnicity and level of deprivation is shown in Figure 3. Girls (OR 2.56 95% CI [2.37, 2.77], *p*‐value < 0.001), gender diverse (OR 4.14 95% CI [3.14, 5.44], *p*‐value < 0.001) and gender non‐disclosing students (OR 2.25 95% CI [1.85, 2.73], *p*‐value <0.001) were all more likely than boys to desire weight support. There was no evidence of any variation in relation to ethnicity or deprivation. Compared to Year 7 (age 11–12), there was evidence of modestly higher desire for weight‐loss support in older children (Table 2).

**TABLE 2:** Mutually adjusted odds ratios from logistic regression models of demographic predictors of weight concern and desire for weight management support.

	Weight concern^a^		Desire for weight support^b^		Desire for weight support (adjusted for weight concern)^c^	
	Odds ratio (95% CI)	*p*‐value	Odds ratio (95% CI)	*p*‐value	Odds ratio (95% CI)	*p*‐value
Weight concern					12.43 (9.26, 16.68)	<0.001
Y07	Ref		Ref		Ref	
Y08	1.14 (1.07, 1.21)	<0.001	1.14 (1.01, 1.28)	0.020	1.07 (0.94, 1.23)	0.290
Y09	1.23 (1.16, 1.31)	<0.001	1.19 (1.05, 1.33)	0.004	1.07 (0.94, 1.23)	0.300
Y10	1.21 (1.12, 1.31)	<0.001	1.20 (1.06, 1.35)	0.004	1.04 (0.89, 1.22)	0.570
Y11	1.32 (1.22, 1.43)	<0.001	1.11 (0.98, 1.24)	0.115	0.85 (0.74, 0.98)	0.030
Y12	1.38 (1.25, 1.52)	<0.001	1.26 (1.08, 1.47)	0.004	1.02 (0.86, 1.22)	0.850
Y13	1.42 (1.26, 1.60)	<0.001	1.08 (0.91, 1.29)	0.375	0.79 (0.65, 0.97)	0.020
Gender boy	Ref		Ref		Ref	
Gender girl	3.56 (3.36, 3.78)	<0.001	2.56 (2.37, 2.77)	<0.001	1.40 (1.27, 1.55)	<0.001
Gender diverse	8.25 (6.78, 10.03)	<0.001	4.14 (3.14, 5.44)	<0.001	1.34 (0.90, 1.98)	0.140
Gender non‐disclosing	3.70 (3.23, 4.25)	<0.001	2.25 (1.85, 2.73)	<0.001	1.35 (1.05, 1.74)	0.020
White ethnicity	Ref		Ref		Ref	
Mixed/multiple ethnicity	0.90 (0.81, 0.99)	0.023	1.09 (0.94, 1.28)	0.254	1.16 (0.96, 1.41)	0.110
Asian ethnicity	0.83 (0.76, 0.89)	<0.001	1.03 (0.92, 1.16)	0.555	1.08 (0.96, 1.22)	0.200
Black ethnicity	0.65 (0.59, 0.72)	<0.001	1.11 (0.94, 1.29)	0.258	1.40 (1.15, 1.71)	0.001
Arab ethnicity	0.61 (0.51, 0.72)	<0.001	0.97 (0.74, 1.28)	0.855	1.40 (0.99, 2.00)	0.050
Other ethnicity	0.98 (0.84, 1.15)	0.747	1.22 (0.97, 1.55)	0.112	1.21 (0.92, 1.59)	0.170
No response	0.84 (0.80, 0.89)	<0.001	1.01 (0.92, 1.11)	0.782	1.11 (0.98, 1.24)	0.100
Deprivation	3.00 (2.24, 4.03)	<0.001	1.43 (0.84, 2.43)	0.113	1.65 (1.20, 2.26)	0.001

*Note*: All models are mutually adjusted for demographic covariates, with school ID included as a random effect.

^a^ Association between demographic variables and *weight concern*.

^b^ Association between demographic variables and *desire for weight management* *support* (unadjusted for weight concern).

^c^ Association between demographic variables and *desire for weight management support*, mutually adjusted for both demographics and weight concern.

### Desire for weight support as a function of weight concern

Increased weight concern was associated with a higher likelihood of desiring weight support across the full sample (OR 12.43, 95% CI [9.26, 16.68], *p* < 0.001). However, this effect varied significantly by subgroup. After adjusting for weight concern, adolescents of Black ethnicity had a higher likelihood of desiring support compared to their White peers (OR 1.40, 95% CI [1.15, 1.71], *p* < 0.001). Students in Year 11 (OR 0.85, 95% CI [0.74, 0.98], *p* = 0.03) and Year 13 (OR 0.79, 95% CI [0.65, 0.97], *p* = 0.02) were significantly less likely than those in Year 7 to want support and, adolescents from the most deprived areas showed greater desire for support compared to the least deprived (OR 1.65, 95% CI [1.20, 2.26], *p* = 0.001). Girls (OR 1.40, 95% CI [1.27, 1.55], *p* < 0.001) and gender non‐disclosing respondents (OR 1.35, 95% CI [1.05, 1.74], *p* = 0.02) were more likely than boys to desire support, even after accounting for their level of weight concern.

**FIGURE 4:**
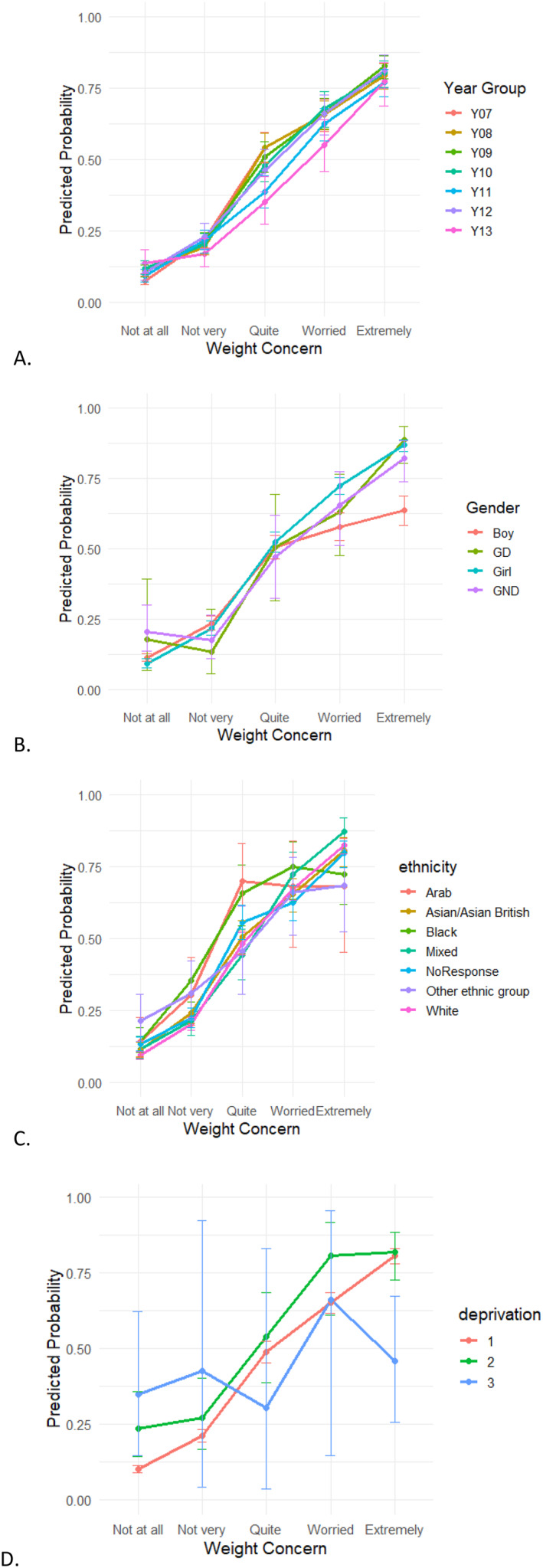
Predicted Probability of desiring weight support by weight concern and (A) gender, (B) ethnicity, (C) year group, (D) level of deprivation.

Using single‐interaction models, we examined how demographic factors moderated the relationship between weight concern and desire for weight support (Figure 4). The association was significantly stronger among girls (OR = 3.35, 95% CI [2.70, 4.16], *p* < 0.001) and gender diverse young people (OR = 2.46, 95% CI [1.02, 5.94], *p* = 0.047) compared to boys. In contrast, weight concern had a significantly weaker association with desire for support among those of Black (OR = 0.46, 95% CI [0.30, 0.71], *p* < 0.001), Arab (OR = 0.39, 95% CI [0.18, 0.86], *p* = 0.018), and Other (OR = 0.28, 95% CI [0.15, 0.50], *p* < 0.001) ethnicities, and among participants who did not disclose their ethnicity (OR = 0.64, 95% CI [0.49, 0.82], *p* < 0.001), relative to those of White ethnicity. A weaker association between weight concern and desire for support was observed in Year 13 students (OR = 0.59, 95% CI [0.38, 0.92], *p* = 0.02) and in those from the most deprived group (OR = 0.10, 95% CI [0.02, 0.44], *p* = 0.002), compared to their respective reference groups.

## DISCUSSION

In a large general health and wellbeing survey of adolescents, 41% expressed some level of weight concern. Weight concern increased with age, White ethnicity and increased levels of deprivation. Girls, gender non‐disclosing and particularly gender diverse young people were all much more likely to report weight concern then boys. In addition, one third of survey respondents reported a desire for weight loss support and this increased slightly with age but with little variation by ethnicity or deprivation. Girls, gender non‐disclosing and gender diverse young people were all more likely than boys to want support. Weight concern was strongly associated with a desire for weight management support.

The strong relationship between girls and increased weight concern is well documented in the literature (Blundell et al., 2024; Creese et al., 2023; Ferreiro et al., 2014) however less is known about the relationship between weight concern and gender minority groups. A recent literature review (Grammer et al., 2019) found no strong evidence of an association between higher weight status and gender minority status; however, it noted that hormone therapy among gender minority individuals is often associated with weight gain. In contrast, the relationship between gender minority status and eating disorders has been more widely studies, with several studies reporting higher rates of eating disorders and body dissatisfaction among gender minorities (Jones et al., 2018; Sampson, 2026). These concerns may extend beyond weight alone, encompassing issues related to body shape, gender expression, and anxieties surrounding pubertal development. It is important to note that the survey question used in this study captured concerns about weight and/or shape. As such, responses may reflect distress related more strongly to body shape than weight in this population. Nevertheless, evidence suggests that weight‐related perceptions remain closely intertwined with gender identity and affirmation and that these are often linked to body weight among gender minority individuals (Cusack et al., 2022).

Girls, gender non‐disclosing, and gender diverse adolescents were more likely to express a desire for weight loss support. This finding appears to be closely associated with higher levels of weight concern in these groups. However, given that boys typically exhibit higher rates of overweight and obesity than girls (England, 2024), the observed differences in support‐seeking are unlikely to be explained by weight status. Previous studies have reported that girls are generally more likely than boys to perceive and report concerns about increased weight (Wardle & Johnson, 2002).

Participants who reported weight concern had 12 times higher odds of desiring weight‐related support. In the main effects model, girls and gender non‐disclosing young people were significantly more likely than boys to desire weight support. However, after accounting for weight concern, girls were only slightly more likely to desire weight support than boys, suggesting that weight concern is the major explaining factor in why girls are more likely to desire support. When interaction terms were included, both girls and gender diverse adolescents showed a significantly stronger association between weight concern and desire for support compared with boys, while no significant difference was observed for gender non‐disclosing participants.

This analysis found that weight concern increased with both age and socioeconomic deprivation, patterns that reflect established trends in both adolescent weight status and eating disorders (England, 2024; Sampson, 2026). Weight concern was highest among adolescents from White ethnic backgrounds, despite national data indicating that young people from these groups have the lowest prevalence of overweight and obesity by ethnicity (England, 2024). Eating disorders are also higher in those of White ethnicity possibly reflecting the link between weight concern and eating disorders (Sampson, 2026). The relationship between ethnicity and weight concern in adolescence remains poorly understood, with existing literature offering inconsistent and sometimes contradictory findings (Kardelen Cakici, 2021; Olson et al., 2020). This highlights the need for further research to explore how cultural norms, body image ideals, and perceptions of weight intersect across different ethnic groups.

Previous research indicates that self‐reported dieting is more common among girls (Houle‐Johnson & Kakinami, 2018; Ojala et al., 2007; Patte & Leatherdale, 2017) and increases with age (Barker & Bornstein, 2010; Harnois‐Leblanc et al., 2021), aligning with the trends observed in this analysis regarding the desire for weight support. Similar studies have also found that weight loss attempts are more frequent among individuals from more deprived backgrounds (Ling et al., 2018) and ethnic minority groups (Ling et al., 2018; Story et al., 2001; Umlauff, 2017). Our analysis found no differences in the desire for weight loss support between ethnic groups and in fact, the interaction model showed that the relationship between weight concern and desire for weight support was strongest in adolescents of White ethnicity. Participants from more deprived backgrounds had higher levels of weight concern but in the interaction model the relationship between weight concern and weight support was mixed. This may suggest that young people from ethnic minority backgrounds or high‐deprivation areas may be more likely to attempt weight loss independently rather than seeking support. Additionally, given that weight tends to be higher among individuals from more socially deprived backgrounds (England, 2024) and we observed no corresponding increase in the desire for weight support with greater deprivation, it suggests that for this sample, the desire for weight support may not be correlated with weight status.

The analysis found that for the oldest age group, year 13, weight concern was negatively correlated with a desire for weight support. This is not something that has previously been reported however we speculate that this may be driven by increased autonomy or previous negative experiences with healthcare. For ethnicity, the interaction models showed the relationship between weight concern and desire for support was significantly stronger for young people identifying as White compared to those from other ethnicities. Those from the most deprived groups have both higher weight concern and a weaker link between weight concern and desire for weight support than their more affluent peers, perhaps reflecting lower expectations of external support.

This study contributes to the limited evidence base on the intersection between weight concern and the desire for weight management support in adolescence. While there is a growing body of literature examining the causes (Kelly et al., 2018; Vuong et al., 2021) and consequences (Blundell et al., 2024; Bornioli et al., 2019; Bornioli et al., 2020) of weight concern among young people, there remains a notable gap in understanding how best to address and manage these concerns. Weight concern occupies a space between physical and mental health, yet it is often inadequately addressed within either domain, potentially leading to unmet needs in both clinical and educational settings. Additionally, the findings suggest that weight concern, rather than weight status alone, is a key driver of adolescents' desire for weight management support. These findings raise important questions about current eligibility criteria for support services, which typically prioritise adolescents who are clinically defined as living with overweight or obesity. As weight status is not necessarily aligned with weight concern or the desire for support, many young people experiencing significant distress related to their weight or body shape may fall outside existing service thresholds. For these adolescents, traditional weight‐loss interventions may not be appropriate. Instead, support may need to focus on psychological wellbeing, body image, or weight‐inclusive approaches that promote overall physical and mental health. Regardless of the form such support takes, the findings suggest that current service models are not adequately meeting the needs of a substantial group of young people. Given the scale of weight concern, and demand for support identified in this study, weight inclusive support should be made more accessible, routine, and embedded within settings where adolescents already spend their time.

A key strength of this study lies in its use of a general questionnaire without a focus on weight and a large, diverse sample of UK adolescents. Given that weight is often considered a shame‐inducing or stigmatised topic (Haqq et al., 2021; Puhl & Lessard, 2020), the use of a non‐identifiable, school‐based survey likely encouraged accurate and complete responses than would be less feasible to collect through clinical or face‐to‐face methods.

Nevertheless, several limitations must be acknowledged. Most notably, the study did not collect objective data on participants' weight status. As such, it is not possible to determine whether reported weight concern was associated with actual weight status, perceived weight status, or broader body image concerns. This limits interpretation of the findings, as weight concern can occur across the weight spectrum and does not necessarily correspond with clinical measures of overweight or obesity. Previous research suggests that adolescents' perceptions of their weight may be more strongly associated with social pressures and sociocultural factors than objectively measured weight status (Voelker et al., 2015). Future studies would benefit from including objective or perceived measures of weight status to better understand how these factors interact.

Only a subset of survey respondents, those who reached that section of the survey and responded to the relevant questions, could be included in the analysis, potentially introducing selection bias. The OxWell survey also excluded certain populations, including young people not present at school on the day of the survey, children excluded from school, those whose parents chose to opt them out of the survey and those attending non‐mainstream schools. Although non‐identifiable, the sensitive nature of weight concern may have led some participants, particularly those with high levels of concern, to skip or avoid related questions, which may have introduced bias into the results (Soneson et al., 2025). Comparisons with population data show that participants from the most socioeconomically deprived backgrounds were markedly under‐represented. However this maybe due to the way deprivation was recorded. While the survey included responses from gender minority groups, these individuals comprised less than 5% of the total sample, which limits the reliability of subgroup analyses and may reduce statistical power.

The cross‐sectional design of this study means that causal relationships cannot be inferred. While associations between weight concern and desire for weight support were observed, it is not possible to determine the directionality of these relationships. Additionally, all data in the OxWell Survey were self‐reported, which introduces the potential for reporting bias and means answers may not be fully generalisable to the adolescent population. Adolescents' answers may have been influenced by social desirability or limited self‐awareness regarding their weight‐related attitudes and behaviours. Finally, the items used to assess weight concern and desire for weight support were not drawn from validated measures, raising the possibility of measurement bias.

## CONCLUSION

This study shows that weight concern is very common among UK adolescents, especially among girls, gender diverse young people, and those from White ethnic backgrounds. About one in three students expressed a desire for weight management support, a need strongly associated with concerns about their weight. Current weight management services, which are typically restricted to young people with overweight or obesity, are unlikely to meet the needs of those whose primary difficulty is weight concern itself. Because weight concern is a psychological construct that can occur independently of weight status, and was strongly associated with desire for support in this study, this study highlghts an urgent need for interventions that explicitly address the mental‐health dimensions of body image and weight‐related distress.

## AUTHOR CONTRIBUTIONS

**Melissa Little**: Writing—original draft; conceptualization; methodology; writing—review and editing; formal analysis. **Mina Fazel**: Conceptualization; funding acquisition; writing—review and editing; methodology; supervision. **Susan A. Jebb**: Conceptualization; writing—review and editing; supervision; methodology; data curation. **Sarah Wane**: Conceptualization; writing—review and editing; methodology; supervision. **Oxwell Study Team**: Conceptualization; funding acquisition. **Paul Aveyard**: Conceptualization; methodology; writing—review and editing; formal analysis; supervision; data curation.

## CONFLICT OF INTEREST STATEMENT

The authors declare no conflicts of interest.

## ETHICAL CONSIDERATION

Adolescent consent and assent was obtained through the survey platform (as described in the study protocol) before taking part in this study. The study was approved by the Research Ethics Committee at the University of Oxford (R62366) on the 19th of January 2023.

## Data Availability

M.L. had full access to all the data in the study and accepts responsibility to submit for publication. The data that support the findings of this study are available from the corresponding author upon reasonable request. Researchers may access the data by applying through the BrainWaves Data Portal (https://brainwaveshub.org/for‐research/) where applications are reviewed to ensure appropriate use. Further details, including the full list of questions, study protocol, and other supporting materials, are available via the OxWell project's Open Science Framework page: https://osf.io/sekhr/.
